# Supplementary figures and images for: Glutaredoxin regulation of primary root growth is associated with early drought stress tolerance in pearl millet
Source: eLife. 2024 Jan 31;12:RP86169. doi: 10.7554/eLife.86169 (PMC10945517; doi:10.7554/eLife.86169)

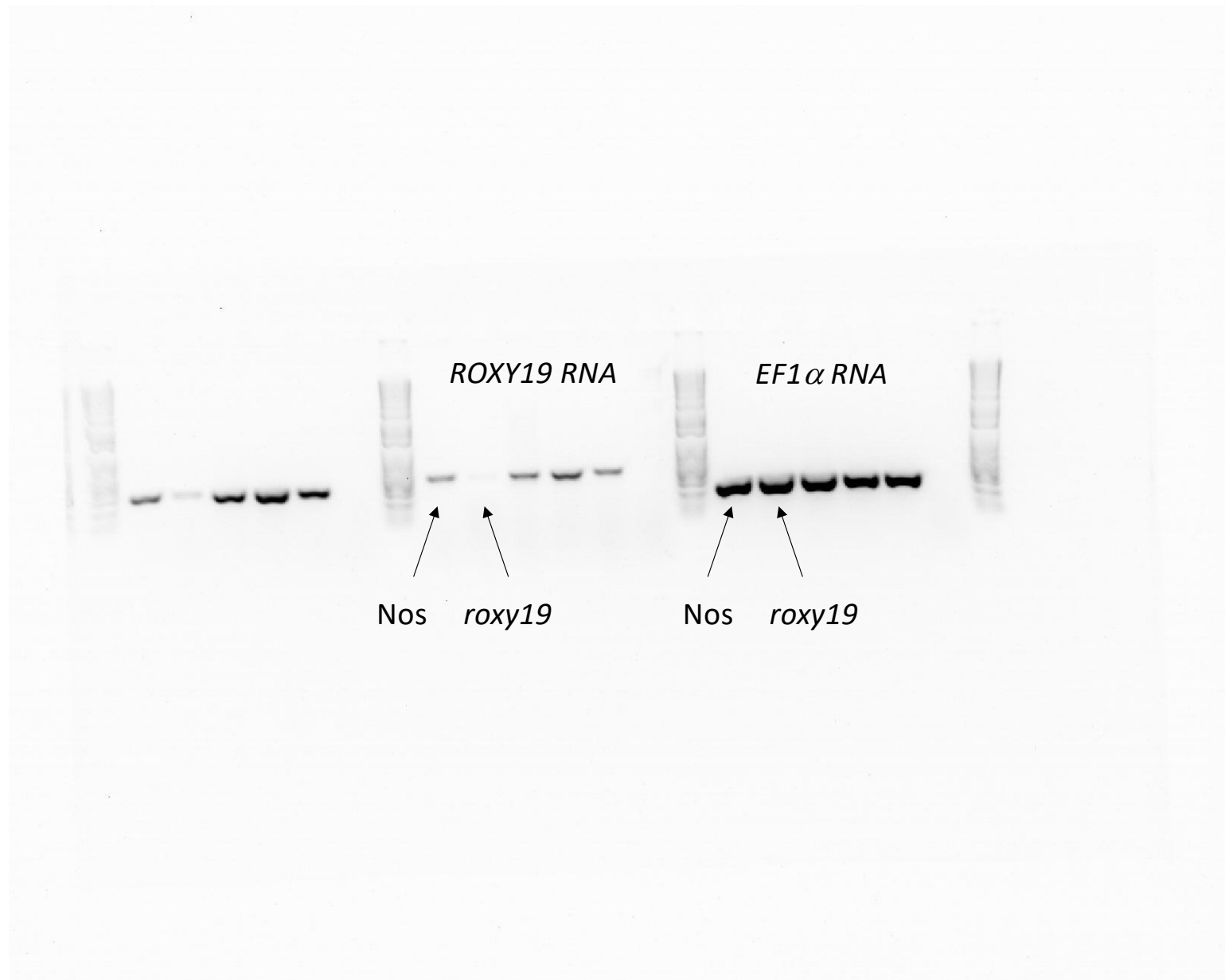

Supplement: Figure 4—source data 1. — No ROXY19 expression was detected in the roxy19 mutant in contrast to the corresponding wild-type background. [file elife-86169-fig4-data1.zip › Figure 4 source data/Figure 4 - source with annotation.pdf]

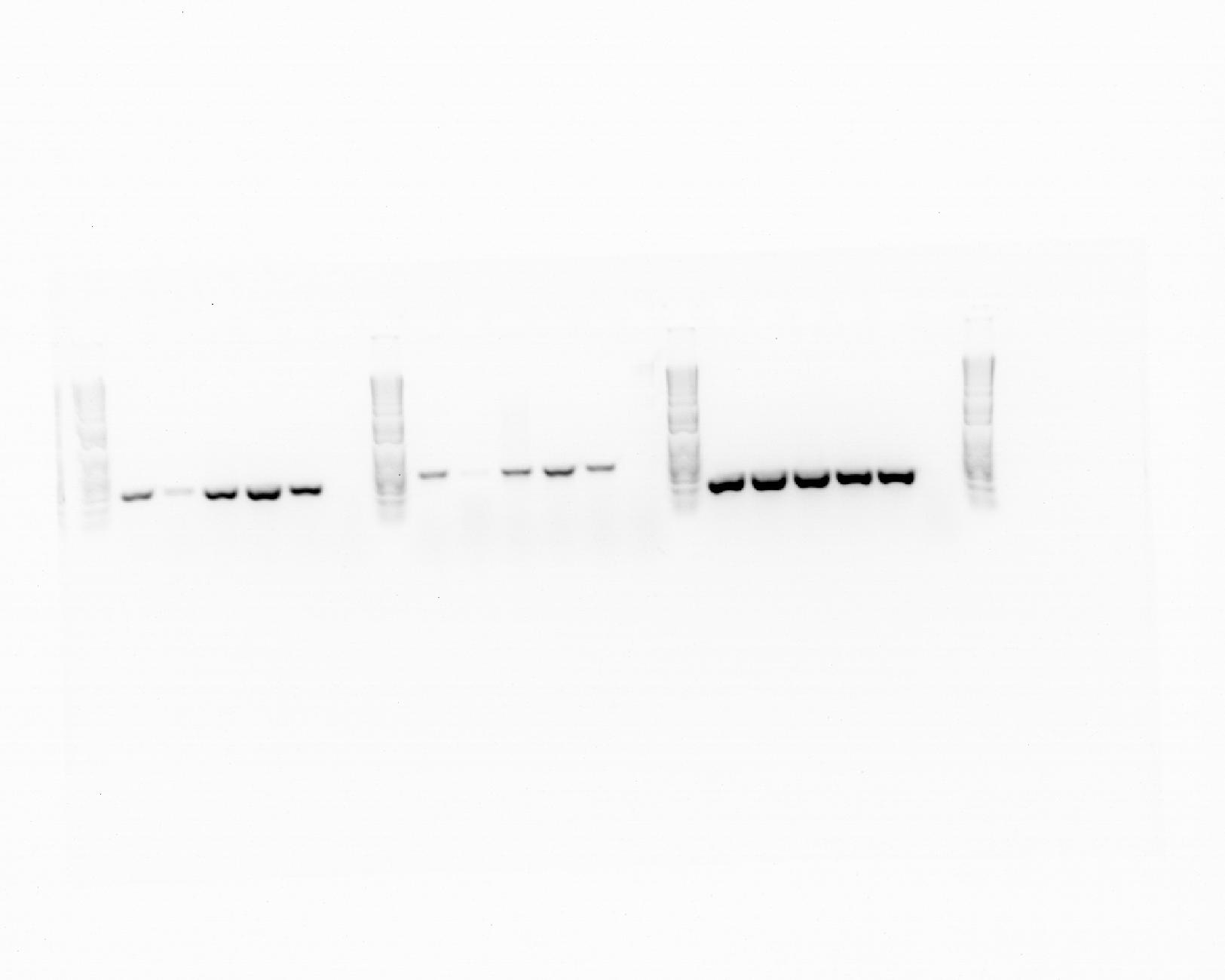

Supplement: Figure 4—source data 1. — No ROXY19 expression was detected in the roxy19 mutant in contrast to the corresponding wild-type background. [file elife-86169-fig4-data1.zip › Figure 4 source data/RT-PCR raw picture.jpg]
