# Supplementary material for: Glutaredoxin regulation of primary root growth is associated with early drought stress tolerance in pearl millet
Source: eLife. 2024 Jan 31;12:RP86169. doi: 10.7554/eLife.86169 (PMC10945517; doi:10.7554/eLife.86169)
Supplement: Supplementary file 1. [file elife-86169-supp1.zip › Table S1.pdf]

Table S1. Drought-tolerance related traits in selected inbred lines grown in field conditions in 2018 and 2020. After sowing, seeds were irrigated with 30 mm of water to allow germination and plants grew for 42 days without any further irrigation. Plant height, stay-green and survival were measured at 42 days after sowing while photosynthesis related traits (FvFm: maximum quantum efficiency of photosystem II; PI: performance index of photosynthesis) were measured at 32 days after sowing. Root length was measured 6 days after germination on a paper-based hydroponic system in growth chamber. Numbers indicate mean ± se.

	Line	Height (cm)	Stay-Green (%)	Survival (%)	FvFm	PI	Root length (mm)
2018	1	16.33 ± 2.62	39.37 ± 2.43	28.67 ± 14.76	0.76 ± 0.03	0.82 ± 0.04	61.05 ± 20.12
	69	23.88 ± 7.25	40.97 ± 6.52	10.48 ± 5.50	0.64 ± 0.19	0.99 ± 0.04	75.65 ± 21.39
	127	17.11 ± 2.97	40.80 ± 4.29	37.83 ± 15.58	0.69 ± 0 .00	0.95 ± 0.08	74.53 ± 16.13
	241	21.97 ± 0.90	41.18 ± 3.40	23.35 ± 18.57	0.73 ± 0.03	0.95 ± 0.26	
	249	17.35 ± 4.98	38.36 ± 8.38	28.29 ± 17.29	0.76 ± 0.02	1.25 ± 0.27	66.35 ± 14.60
	263	41.37 ± 5.41	35.61 ± 2.67	58.94 ± 21.17	0.76 ± 0.01	1.07 ± 0.07	107.21 ± 10.89
	337	27.94 ± 5.57	43.37 ± 6.29	47.23 ± 13.94	0.77 ± 0.01	1.33 ± 0.20	149.30 ± 10.04
	343	35.10 ± 6.65	40.40 ± 1.03	22.53 ± 13.75	0.77 ± 0.01	1.16 ± 0.27	79.91 ± 20.59
	344	26.69 ± 5.90	46.21 ± 9.23	31.48 ± 11.94	0.70 ± 0.08	1.32 ± 0.30	167.57 ± 12.92
2020	3	28.67 ± 5.75	60.87 ± 4.66	64.48 ± 9.21	0.74 ± 0.01	2.95 ± 0.55	58.94
	55	31.54 ± 8.64	56.53 ± 7.22	58.07 ± 13.18	0.74 ± 0.02	3.97 ± 0.79	61.21 ± 12.75
	69	19.58 ± 4.14	51.92 ± 7.28	76.11 ± 18.93	0.73 ± 0.01	3.21 ± 0.33	75.65 ± 21.39
	81	24.28 ± 1.37	54.20 ± 9.02	41.40 ± 8.83	0.76 ± 0.01	4.55 ± 1.29	136.53 ± 21.19
	132	28.67 ± 2.23	56.15 ± 2.01	66.93 ± 5.03	0.72 ± 0.02	3.34 ± 0.22	68.70 ± 12.99
	249	19.83 ± 4.09	56.08 ± 6.79	70.43 ± 18.91	0.72 ± 0.03	2.85 ± 1.12	66.35 ± 14.60
	263	24.42 ± 4.31	61.88 ± 4.94	70.83 ± 15.35	0.74 ± 0.01	4.04 ± 0.56	107.21 ± 10.89
	337	30.63 ± 4.54	61.65 ± 2.70	59.53 ± 10.56	0.74 ± 0.01	3.44 ± 0.27	149.30 ± 10.04
	343	30.67 ± 6.38	56.27 ± 4.95	58.75 ± 4.93	0.74 ± 0.01	3.68 ± 0.67	79.91 ± 20.59
	344	27.42 ± 4.06	60.18 ± 4.10	81.51 ± 14.29	0.75 ± 0.01	4.76 ± 0.51	167.57 ± 12.92
p-values	*Line*	*	*0.84*	*0.64*	*0.54*	*0.29*	***
	Year	*0.31*	***	***	*0.86*	***	*0.73*
	Line x Year	*0.27*	*0.88*	*0.28*	*0.34*	*0.28*	*0.98*
lsmeans	69	21.8	46	43.3	0.693	2.19	75.7
	249	18.6	47.2	49.4	0.741	2.05	66.3
	263	33.1	49.3	64.9	0.755	2.58	107.2
	337	29.3	52.5	53.4	0.754	2.36	149.3
	343	32.9	49.1	40.6	0.759	2.52	79.9
	344	27.1	53.2	56.5	0.729	3.04	167.6
