# Supplementary material for: Glutaredoxin regulation of primary root growth is associated with early drought stress tolerance in pearl millet
Source: eLife. 2024 Jan 31;12:RP86169. doi: 10.7554/eLife.86169 (PMC10945517; doi:10.7554/eLife.86169)
Supplement: Supplementary file 1. [file elife-86169-supp1.zip › Table S2.pdf]

Table S2. GWAS *p*-values for the significant SNP markers identified through LFMM (ridge) using four other GWAS methods

Chr.	Position (Mbp)	Allele Freq REF	Allele Freq ALT	GWAS p-values[1]				
				ANOVA	EMMA	MLM	LFMM (LEA)	LFMM (ridge)
1	7.848	0.828	0.172	ns	ns	ns	***	***
1	30.561	0.910	0.090	***	***	**	**	***
1	52.188	0.730	0.270	**	**	**	***	***
1	65.933	0.475	0.525	ns	ns	ns	***	***
1	109.737	0.893	0.107	*	*	*	**	***
1	130.348	0.934	0.066	*	*	*	**	***
1	231.244	0.754	0.246	*	*	*	***	***
1	231.246	0.672	0.328	*	*	*	***	***
1	231.265	0.295	0.705	*	ns	*	***	***
1	247.636	0.836	0.164	***	***	**	***	***
1	247.637	0.852	0.148	**	**	**	***	***
1	253.567	0.648	0.352	**	*	*	***	***
2	40.574	0.164	0.836	***	ns	***	***	***
2	60.654	0.926	0.074	**	**	**	*	***
2	85.902	0.656	0.344	***	***	**	***	***
2	92.005	0.689	0.311	*	*	ns	***	***
2	113.747	0.631	0.369	*	*	*	***	***
3	1.657	0.934	0.066	**	*	*	*	***
3	8.847	0.951	0.049	**	**	**	ns	***
3	14.107	0.484	0.516	***	ns	**	***	***
3	14.107	0.459	0.541	**	ns	**	***	***
3	14.124	0.254	0.746	***	ns	**	***	***
3	14.192	0.164	0.836	ns	ns	ns	***	***
3	14.602	0.672	0.328	**	*	*	***	***
3	53.909	0.566	0.434	**	**	*	***	***
3	224.557	0.443	0.557	***	ns	*	***	***
3	225.059	0.762	0.238	*	*	ns	***	***
3	264.082	0.934	0.066	*	*	*	*	***
3	267.439	0.918	0.082	***	***	**	**	***
3	294.825	0.041	0.959	**	ns	**	ns	***
4	4.566	0.230	0.770	**	ns	*	***	***
4	16.258	0.861	0.139	ns	ns	ns	***	***
4	45.631	0.869	0.131	*	**	**	**	***
4	113.503	0.770	0.230	*	**	*	***	***
4	121.206	0.787	0.213	*	**	**	***	***
4	122.223	0.820	0.180	***	***	**	***	***
4	124.508	0.893	0.107	**	***	***	**	***
5	97.383	0.836	0.164	*	*	*	***	***
6	36.876	0.713	0.287	***	**	**	***	***
6	52.098	0.713	0.287	***	***	**	***	***
6	157.606	0.082	0.918	*	ns	ns	**	***
7	10.770	0.787	0.213	ns	*	*	***	***
7	10.819	0.918	0.082	**	**	*	**	***
7	34.533	0.967	0.033	*	*	*	ns	***
7	149.890	0.697	0.303	**	**	*	***	***

[1] Analysis of variance (ANOVA), Efficient Mixed Model Analysis (EMMA), Mixed Linear Model (MLM) and Latent Factor Mixed Model using the algorithm MCMC (LFMM LEA). *** P-value ≤ 0.0001, ** P-value ≤ 0.001, * P-value ≤ 0.01, *ns* not significant
