# Supplementary material for: Glutaredoxin regulation of primary root growth is associated with early drought stress tolerance in pearl millet
Source: eLife. 2024 Jan 31;12:RP86169. doi: 10.7554/eLife.86169 (PMC10945517; doi:10.7554/eLife.86169)
Supplement: Supplementary file 1. [file elife-86169-supp1.zip › Table S3.pdf]

Table S3. Significant genomic regions identified by Bulk Segregant Analysis (BSA) for primary root length at the 95% confidence interval.

	Chr.	Peak position[1] (Mbp)	Region range[2] (Mbp)	Region length (Mbp)	Number sig SNPs
RL1.1	1	52.57	31.21 – 60.57	29.36	7
RL1.2	1	176.47	165.42 – 195.36	29.94	71
RL1.3	1	215.85	196.38 – 239.10	42.72	185
RL6.1	6	89.99	4.55 – 101.68	97.13	695
RL6.2	6	116.91	108.03 – 125.64	17.61	37
RL6.3	6	201.08	176.52 – 240.48	65.79	290

[1] Position of the most significant SNP in the region range

[2] Limits of the significant region considering the overlapping confidence interval of significant markers in the region
