# Supplementary material for: Glutaredoxin regulation of primary root growth is associated with early drought stress tolerance in pearl millet
Source: eLife. 2024 Jan 31;12:RP86169. doi: 10.7554/eLife.86169 (PMC10945517; doi:10.7554/eLife.86169)
Supplement: Supplementary file 1. [file elife-86169-supp1.zip › Table S4.pdf]

Table S4. Common GWAS and BSA Marker trait associations for primary root length.

Chr.	Position (Mbp)	Allele Freq REF	Allele Freq ALT	GWAS $-\log_{10}$ (*pvalue*)	BSA sig region (95% CI)
1	52.188	0.730	0.270	4.623	*RL1.1*
1	231.244	0.754	0.246	4.200	*RL1.3*
1	231.246	0.672	0.328	4.531	
1	231.265	0.295	0.705	8.413	
6	36.876	0.713	0.287	4.020	*RL6.1*
6	52.098	0.713	0.287	4.018	
