# Supplementary material for: Glutaredoxin regulation of primary root growth is associated with early drought stress tolerance in pearl millet
Source: eLife. 2024 Jan 31;12:RP86169. doi: 10.7554/eLife.86169 (PMC10945517; doi:10.7554/eLife.86169)
Supplement: Supplementary file 1. [file elife-86169-supp1.zip › Table S5.pdf]

Table S5. Lines selected for field trials. Average root length represents the value observed in paper growth system in the original phenotyping performed for GWAS.

Line	Average root length cm^{-1}
ICML-IS 11001	61.05
ICML-IS 11002	59.94
ICML-IS 11047	75.65
ICML-IS 11108	172.18
ICML-IS 11155	66.35
ICML-IS 11165	107.21
SL 2	149.3
SL 4	79.91
SL 5	167.57
